# A Quality Improvement Initiative to Increase Central Line Maintenance Bundle Compliance through Nursing-led Rounds

**DOI:** 10.1097/pq9.0000000000000515

**Published:** 2022-01-21

**Authors:** Maria C. Hugo, Rheannon R. Rzucidlo, Lauren M. Weisert, Isaac Parakati, Sangeeta K. Schroeder

**Affiliations:** From the * Department of Nursing, Ann & Robert H. Lurie Children’s Hospital of Chicago, Chicago, Ill.; † Center for Quality and Safety, Ann & Robert H. Lurie Children’s Hospital of Chicago, Chicago, Ill.; ‡ Data Analytics and Reporting, Ann & Robert H. Lurie Children’s Hospital of Chicago, Chicago, Ill.; § Department of Pediatrics, Ann & Robert H. Lurie Children’s Hospital of Chicago, Chicago, Ill.; ¶ Department of Pediatrics, Northwestern University, Feinberg School of Medicine, Chicago, Ill.

## Abstract

**Introduction:**

Improvements in maintenance bundle compliance around central line-associated bloodstream infections (CLABSIs) lack standardization. The objective was to implement a formalized nursing-led rounding process, Rounds for Influence, with a goal of 12 rounds/wk on each inpatient unit and Ambulatory Infusion Center, achieving > 90% maintenance bundle compliance.

**Methods:**

Nurses served as peer “influencers” to perform rounds. The CLABSI prevention team created three comprehensive rounding tools (line access, dressing change/port needle insertion, and cap change) on a digital platform. The team designed these tools to assess clinical competence for maintenance bundle components and implemented nine plan-do-study-act cycles throughout the study period.

**Results:**

Influencers completed 191 rounds after the first month of implementation, resulting in a 264.2% increase from the baseline of 52.5 rounds per month. Over the 2.5 years postimplementation, rounds resulted in 7836 total observations. Maintenance bundle compliance decreased from 86.9% (centerline value from November 2017 to September 2018) to 40.8% after the first month of implementation. Compliance increased iteratively (two separate centerline shifts) to a current centerline value of 87.1%. The CLABSI 12-month cumulative standardized infection ratio (SIR) was 0.9 in November 2017 and dropped to 0.53 in June 2021.

**Conclusion:**

Implementing a formalized nursing-led rounding process led to increased maintenance bundle compliance, decreased CLABSI SIR, and is an integral part of nursing practice. Given this success, there is interest from other hospital-acquired condition improvement teams in applying this rounding practice to their improvement work.

## INTRODUCTION

Central line-associated bloodstream infections (CLABSIs) are costly, extend the length of stay, and contribute to 30,000 deaths per year.^1–3^ Pediatric CLABSIs have an attributable length of stay of 19–21 days and an estimated cost of $55,000–$69,000 per infection.^3^ CLABSIs are preventable by implementing evidence-based insertion and maintenance bundles (MBs).^4–7^ National collaboratives, including the Children’s Hospital Association and Solutions for Patient Safety, advocate using nursing audits to improve MB compliance.^8^ Nurses perform audits through various mechanisms that incorporate transparency of bundle compliance and real-time feedback.

**Fig. 1.**
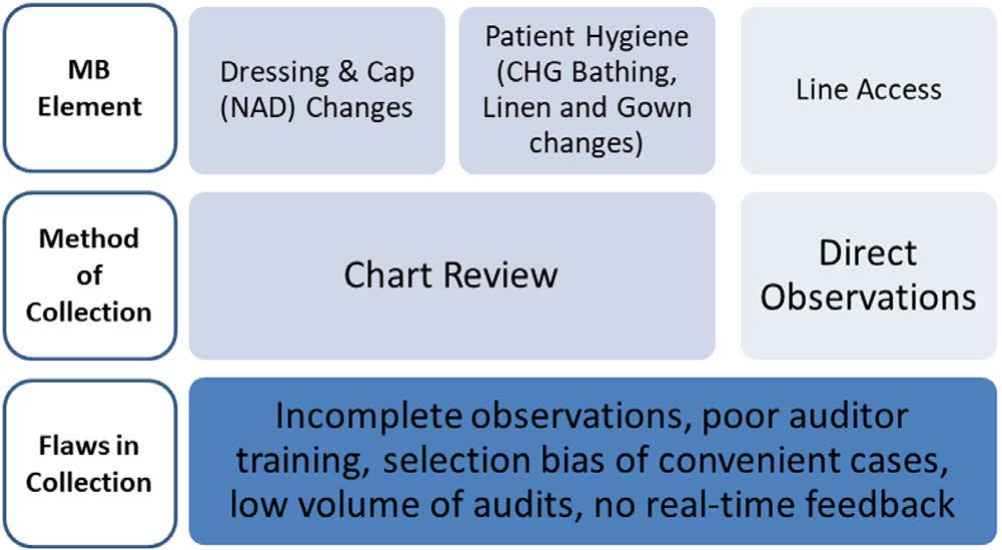
MB compliance data collection methods. MB, maintenance bundle; NAD, needleless access device.

The CLABSI prevention team introduced these bundles in 2012. In 2014, the team implemented an improvement initiative to optimize MB compliance using traditional data collection methods (Fig. 1). Our institution measures CLABSIs via a standardized infection ratio (SIR) as defined by the National Healthcare Safety Network. The SIR is the actual number over the expected number of CLABSIs.^9^ Initial results of this improvement initiative showed a reduction of total CLABSIs with a 12-month cumulative SIR of 0.5 (half the number expected) in December 2015. The SIR increased to 0.9 by November 2017. In addition, local investigation of CLABSIs revealed MB noncompliance as the most frequent risk factor for infection.

To address flawed audit strategies (Fig. 1), the team implemented a formalized nursing-led MB observation process, called Rounds for Influence (RfI), to improve MB element assessments and compliance. This high-quality, high-frequency process focuses on MB elements of line access, dressing and cap changes, and daily hygiene.^4,8,10^ Unlike the traditional auditing process, RfI necessitates that the observer watches each element of the MB and stops the procedure in real-time to educate for protocol breaks which focuses the culture on learning and continuous improvement. We chose to call these observations “rounds” to promote the collaboration and real-time feedback within each observation versus the previous passive process of audits.

The target of RfI is to obtain 12 rounds/wk on each inpatient unit and ambulatory infusion center to achieve > 90% MB compliance.

## METHODS

This institution is a 364-bed freestanding quaternary care, urban, academic children’s hospital with seven inpatient units, all of which are in scope, including the ambulatory infusion center. These units include pediatric intensive care, comprehensive cardiac care, neonatal intensive care, stem cell transplant/hematology/oncology, and three surgical and medical acute care units. Institutional Review Board approval is not required since this initiative aims to implement best practices and not discover novel therapies.

### Forming and Training the Team

Members of the CLABSI prevention team (RfI leaders) recruited 13 registered nurses (RNs) as peer “influencers” to perform rounds in their respective units. The RfI leaders selected candidates based on clinical experience, previous quality improvement work, and their ability to provide real-time peer feedback. Some of these influencers already functioned as a clinical quality coordinator (CQC) who manages their respective units’ safety and quality initiatives.

The RfI leaders created in-depth influencer training including: (1) demonstration of clinical skills facilitated by the hematology/oncology nurse educator; (2) workshop on delivering feedback taught by a leadership development specialist; (3) scripting explaining RfI to patients and families created by the director of Patient Family Experience; and (4) training on the web application (Rounds+) where influencers would record their rounds. RfI leaders also facilitated simulations of these methods.

### Creating the Tool

**Fig. 2.**
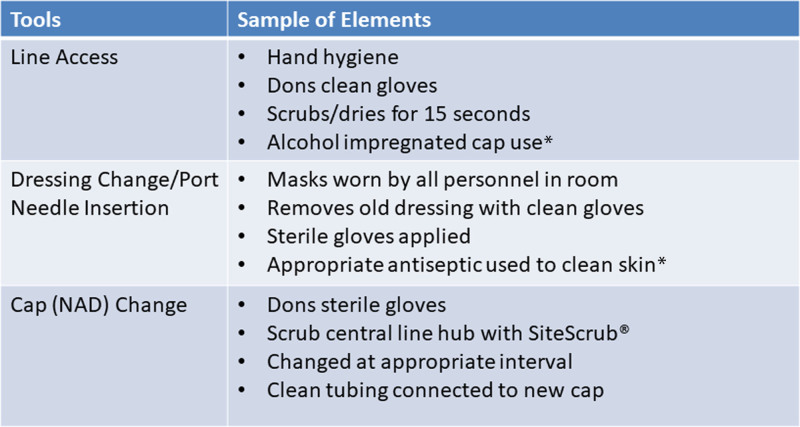
Rounds for influence MB tool detail. MB, maintenance bundle; NAD, needleless access device. *The list of elements shown is not inclusive of all elements.

Rounds+ is a digital patient rounding application licensed by the GetWellNetwork, a digital patient engagement company (GetWellNetwork, Inc., Bethesda, Md.).^11^ RfI leaders created three tools on this digital platform for influencers to use during rounds observing line access, dressing change/port needle insertion, and cap change. The content of each tool came from the institution’s nursing protocol for central line (CL) management and included each element as needed to perform the MB. Each tool contains 20–25 distinct elements, with all tools containing the two patient-hygiene-specific elements of chlorhexidine gluconate bathing and linen/gown changes. Each tool also contains summative questions related to the two other tools to capture the entire MB compliance accurately. For example, the line access tool contains two questions for the influencer to verify the dressing status and the appropriate timing of the cap and dressing changes. Figure 2 shows a sample of elements for each tool. Each element includes the options “yes,” “no,” or “done with correction.” In addition, several elements included an option for “N/A” to account for patient-specific conditions. Rounds+ was already in use throughout the hospital in other capacities, and each unit had a mobile digital device designated for patient rounding. This availability enabled influencers to bring the device to the bedside to perform rounds. Furthermore, influencers would explain the initiative to families if they were present during the RfI process.

### Process of a Round for Influence

The round process begins with influencers identifying which patients on their respective units have a CL and reaching out individually to those patients’ nurses or the unit indicating they are available for RfI. Nurses then notify the influencer to come to observe them when their patient’s next MB element is being performed. During the round, the influencer brings the mobile digital device with the Rounds+ application to document all respective elements observed. The influencer stops the procedure if any protocol breaks occur and offers real-time education to the nurse. When potential breaks in protocol do occur, the element is documented as “done with correction.”

### Implementation

Influencers began performing RfI in November 2018, spending approximately 4 hours/wk to complete 12 rounds/unit each week. The influencers on four units were CQCs who incorporated RfI into their existing schedules. Two units had bedside RNs serving as influencers who used additional time outside of patient care, allotted by departmental leadership. Two units used a combination of bedside RNs and CQCs. Two resource team RNs performed rounds across all units as influencers.

### Studying the Intervention and Plan-do-study-act Cycles

**Fig. 3.**
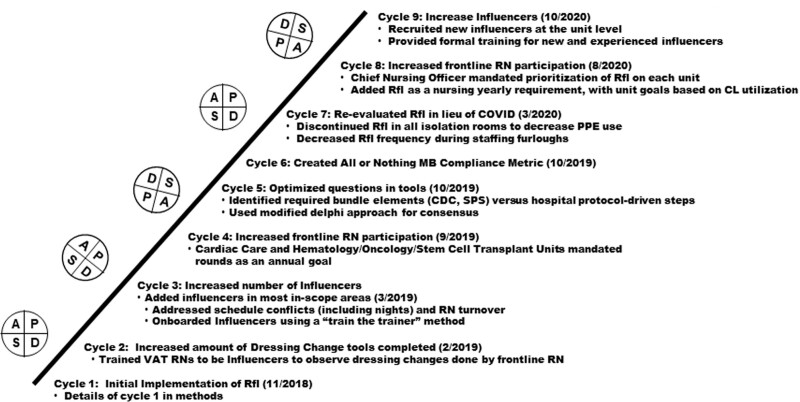
PDSA ramp. CDC, Centers for Disease Control; CL, central line; NAD, needleless access device; PDSA, plan do study act; Rfl, rounds for influence; RN, registered nurse; SPS, solutions for patient safety; VAT, vascular access team.

RfI leaders received updates from influencers during CLABSI improvement meetings every three weeks and during two dedicated check-ins. For the first 12 months of RfI, six PLAN-do-study-act (PDSA) cycles occurred, including implementation. Three additional PDSA cycles occurred over the subsequent 12 months (Fig. 3). These PDSA cycles were a direct result of qualitative and quantitative data. These data included feedback from influencers and frontline nurses, and individual rounds.

Data analysts created a reporting structure to feed Rounds+^11^ data into a weekly spreadsheet report and a web-based analytics tool, Microsoft Power BI. RfI leaders received these weekly spreadsheet reports via email detailing each completed round and individual element compliance. In addition, Microsoft Power BI displayed CLABSI 12-month cumulative SIR, MB compliance, individual element compliance, and rounding frequency in a hospital-wide preventable harm dashboard. Data analysts granted preventable harm dashboard access to members of the CLABSI team, including nurses, physicians, infection preventionists, improvement specialists, and executive sponsors. Unit leadership used the dashboard to direct quality improvement based on individual MB element compliance and the rounding volume on their units.

The primary outcome metric was the CLABSI 12-month cumulative SIR. In addition, RfI leaders analyzed the rounding volume on each unit and MB compliance. The goal of weekly rounds per unit was 12, with the potential for multiple rounds to occur on the same patient. MB compliance was initially defined as the total number of compliant elements divided by the number of observed elements. It revealed compliance of >90% for all three rounding tools, whereas individual elements’ compliance remained well below. Therefore, PDSA cycle 6 (Fig. 3) revised the MB compliance metric to “all-or-nothing” compliance, defined as compliant rounds divided by the total number of observed rounds. The RfI leaders considered a round compliant if every element associated with that round had a compliant response of either “yes” or “N/A.” Noncompliant responses were “no” or “done with correction.” The tools’ element compliance was measured to prioritize improvement endeavors. RfI leaders monitored CLABSI incidence via a 12-month cumulative SIR throughout the PDSA cycles.

RfI leaders also tracked two balancing metrics: a quantitative metric, measurement of the continued use of the previous MB “auditing tool,” and a qualitative metric, analysis of why influencers were unable to perform RfI. RfI leaders collected qualitative data through discussions with influencers and local unit leadership.

The RfI leaders validated each metric in partnership with data analysts via a manual chart review of a convenience sample. Metrics were stratified by patient location, tool type, and time. MB compliance and the rounding volume were monitored over time using statistical process control charts (SAS enterprise guide 7.1; SAS, Cary, N.C.).

During this initiative, organizational leaders identified CLABSI reduction as an area of focus based on strategic goals. This focus led to increased awareness and executive leadership involvement in MB improvement efforts. Some concurrent improvements included the following: a stop sign sticker outside patient rooms to prevent interruptions during sterile procedures, escalation pathway for clinicians to use if patients and families refused CL maintenance care, daily sanitation of high-touch surfaces in patient rooms, chlorhexidine gluconate/alcohol combination device swab trial, and a GetWellNetwork^11^ educational video for patients and families regarding the purpose and care of CLs. These improvements were implemented collectively as a CLABSI toolkit in May 2019.

## RESULTS

**Fig. 4.**
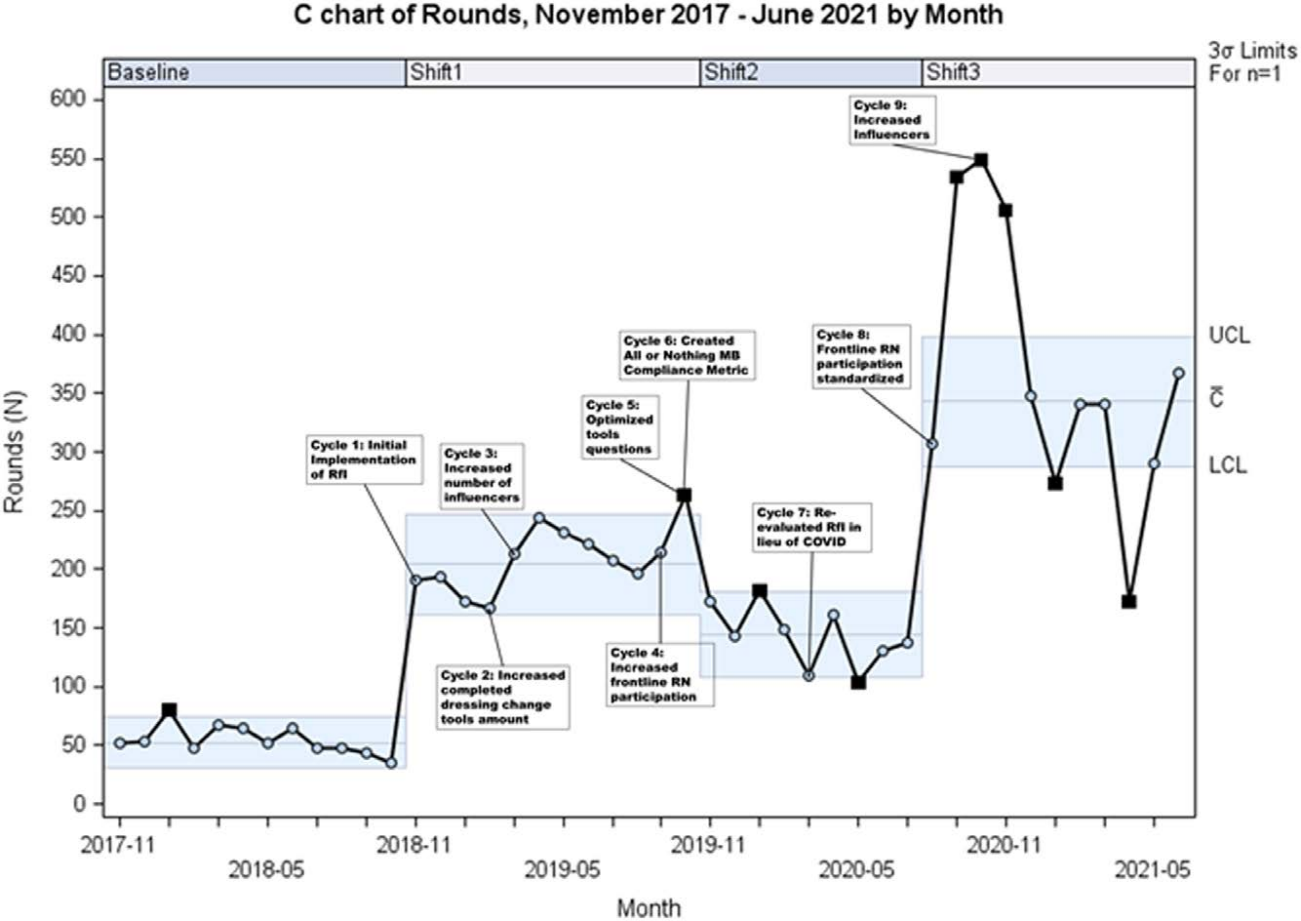
Number of rounds by month from November 2017 to June 2021.

RfI started in November 2018 with a hospital-wide goal of 336 rounds/mo. After the initial month, influencers completed 191 rounds, resulting in a 264.2% increase from the 52.5 rounds/mo baseline. Figure 4 displays a statistical process control chart demonstrating continuous rounding volume improvement from PDSA cycles 1–9. It shows multiple centerline shifts, with the most recent occurring in August 2020 (343 rounds/mo).

PDSA cycles 1–3 increased dressing change tools from 50 (November 2018–January 2019) to 73 (February 2019–April 2019). Likewise, PDSA cycles 4, 8, and 9 resulted in an increase in the number of influencers from 13 (November 2018) to 35 (September 2019) to 88 (October 2020).

The total number of direct observation audits during the 12 months before RfI was 421, only line access audits. An additional 236 chart reviews were done on cap and dressing changes, resulting in 657 entries into the previous auditing system. Over the 2.5-year postimplementation period, influencers completed 7836 RfIs, approximately 10% of all CL days. Line access was the most frequently used tool (n = 4845), accounting for 61.8% of all RFIs. Cap change (n = 1473) comprised 22.2% and dressing change/port needle insertion (n = 1248) comprised 15.9% of RfIs.

**Fig. 5.**
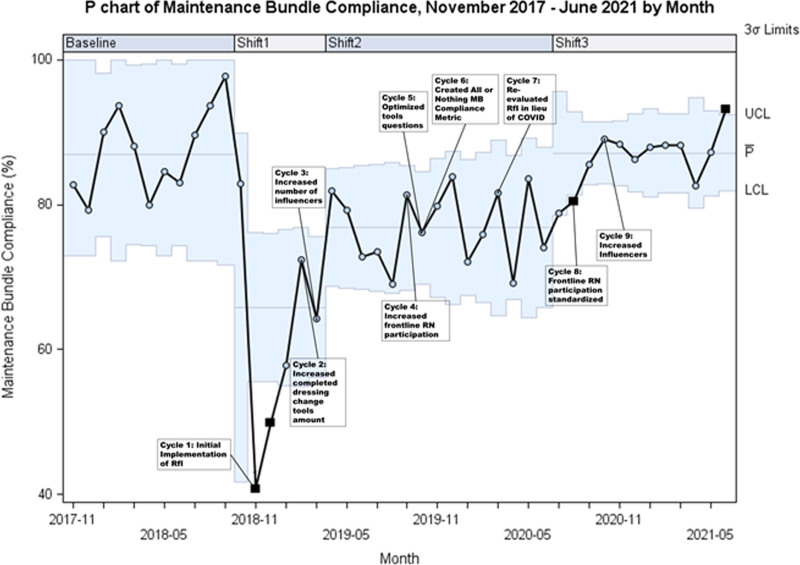
MB compliance by month November 2017 to June 2021.

MB compliance initially decreased from 86.9% (centerline value from November 2017 to September 2018) to 40.8% after the first month of implementation. However, compliance increased iteratively (two separate centerline shifts) throughout the study period with a current centerline value of 87.1% (Fig. 5).

**Fig. 6.**
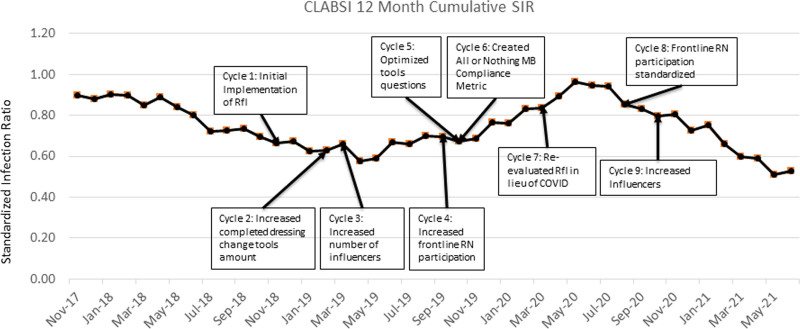
CLABSI 12-month cumulative SIR November 2017 to June 2021.

CLABSI 12-month cumulative SIR started at 0.9 (November 2017) was at its highest point of 0.96 (May 2020), and is currently at 0.53 (June 2021) (Fig. 6).

The qualitative analysis offered several reasons why influencers could not complete targeted rounding volumes: (1) Initially, RNs were hesitant to be observed and avoided participating. (2) Influencers were required to staff units during times of high census and COVID-19-related furloughs. (3) Influencers working as full-time salaried employees had to balance rounding with their weekly workload. (4) Influencers scheduled to obtain rounds in a 4-hour block were not always able to round based on patients’ scheduled CL needs. (5) When multiple patients had simultaneous CL needs, the influencer could only round on one.

## DISCUSSION

Findings demonstrate that implementation of RfI led to a CL shift increase in both overall MB rounds volume and MB compliance and a decrease in the CLABSI SIR. Strengths of this initiative include a standardized rounding approach, data transparency, and diversity of participating units.

Formal training of influencers (see methods: Forming and Training the Team) led to this standardized rounding approach, which increased reliability and validity in data collection. Another strength is real-time education and feedback, allowing immediate intervention to prevent noncompliant MB elements from occurring. Data analysts developed a comprehensive display of RfI data in Power BI. This level of data transparency and access allowed unit leaders and influencers to plan educational and MB improvement endeavors based on real-time observations of barriers to compliance. One example was an initiative to increase hand hygiene compliance in the cardiac care unit based on low compliance data for this element. During rounds, feedback from frontline RNs indicated a need for increased access to hand sanitizer and gloves in the patient rooms. This level of data transparency also allowed senior leadership to hold units accountable to address these barriers. In addition, data were reflective of the diverse group of participating units’ trends and practices. This study breadth led to shared learning across all in-scope areas regarding the operations of RfI and data analysis to drive MB improvements.

Following the implementation of RfI, there was a sharp decrease in reported MB compliance (86.9% to 40%). The RfI leads understood this shift as secondary to RfI’s high-quality, standardized rounding process, which more accurately captured actual MB performance. Throughout the postimplementation period, a gradual increase in compliance occurred secondary to iterative improvements. The subsequent decrease in RfI volume seen in November 2019 aligned with a sudden, unexpected increase in the patient census from an external decrease in city-wide hospital beds. The RfI volume remained low during periods of high census, which lasted until early March 2020. As COVID-19 spread locally, RfI leads paused rounds for patients in isolation to conserve personal protective equipment. Moreover, staffing furloughs, which occurred April 2020 through July 2020, resulted in a pause on all nursing activities that were not direct patient care, including RfI. Once furloughs ended, PDSA cycles 8 and 9 focused on increasing influencers and frontline RN participation. This focus increased the RfI volume, which was a driver in the parallel MB compliance increase, nearing the goal of 90% (Figs. 4 and 5). This increase in MB compliance was a key driver in reducing the 12-month cumulative CLABSI SIR to 0.53 (Fig. 6).

Hermon et al^12^ demonstrated a feedback system helped in ongoing MB compliance. The documentation of MB elements (review of CL necessity, an inspection of the insertion site, asepsis during line access, sterile glove use, and appropriate scrub time) occurred twice daily. This study showed that giving monthly feedback on MB data to the intensive care unit decreased the CLABSI rate and improved MB compliance to >90%.^12^ Our team’s improvements incorporated MB documentation feedback into RfI processes since the tools include questions about the documentation of specific elements. Unit leadership teams accessed Power BI to see weekly data and share this data with their respective teams.

Frith et al^13^ compared traditional audits to another formalized rounding process, called Kamishibai cards (K-cards). K-cards were used to observe MB compliance for dressing status/documentation, tubing timed/dated, hub disinfection, cap status/documentation, and line necessity discussion. The study did not describe the level of detail observed for each of the above components and only included one unit. Only tubing timed/dated had a statistically significant change after the implementation of K-Cards.^13^ Our team’s improvements focused on observing individual steps to perform critical MB skills (line access, cap change, and dressing change) and evaluating overall MB from summative questions described in the Methods section (Creating the tool). This approach resulted in a more complete picture of MB compliance while offering real-time feedback on intricate procedures within the MB.

The CLABSI prevention team recognized differences between observed versus hypothesized outcomes during select periods throughout the initiative. As described above, the CLABSI team anticipated a drop in MB compliance data. Instead, the data demonstrated an unexpected sustained MB compliance of around 90% for all three tools. Therefore, the team restructured the data to represent an all-or-nothing MB compliance metric in October 2019 (PDSA cycle 6). This new metric was retroactively applied to data starting November 2018, which immediately resulted in an all-or-nothing MB compliance of 40.8%. Although this new metric resulted in a call to action for improvement, it also coincided with the city-wide shortage of RNs and subsequent high census period as described above, slowing down the next phase of improvements. Once the organization realigned priorities during COVID-19 and furloughs ended, the team proceeded with PDSA cycles 8 and 9, resulting in increased MB compliance and decreased CLABSI SIR.

A limitation to the MB compliance metric interpretation was the concurrent CLABSI toolkit implementation in May 2019. Although improvement efforts within the toolkit did not directly impact the skills observed during RfI, it is possible that the toolkit itself had a role in MB compliance. Given this was an organizational priority, it was not appropriate to delay implementing the toolkit. A second limitation was the potential for bias during the RfI process, as influencers performed rounds on the skills completed by their peers. To minimize this intrinsic bias, all influencers went through a standardized training process. Last, at the time of implementation of RfI, safety coaches and formal training around error prevention and leadership methods were not implemented across the organization. To address this, the RfI leads worked with leadership development specialists to incorporate training specific to delivering feedback. RfI leads also implemented simulation sessions to augment the influencers’ learning.

Executive sponsorship and realignment of organizational goals provided the resources necessary to limit generalizability, including nursing, data analysts, and technology. Nurses filled the roles of the influencers, which required the support of the chief nursing officer to designate time outside of patient care. The RfI leaders consisted of two nurse managers who incorporated this improvement work into their existing workload. Leadership approved the allocation of data analysts’ time for the MB metrics build in Power BI. The CLABSI prevention team’s use of technology, including a mobile digital device on each unit, the Rounds+ application,^11^ and Power BI, enabled data entry and transparency in real time. These technologies would be an investment if not already in existence at another organization.

## CONCLUDING SUMMARY

In conclusion, implementation of RfI demonstrates an increased MB compliance, decreased CLABSI SIR, and is an integral part of nursing practice at this institution. Having a peer observe skills and provide real-time feedback leads to higher accountability. Given the success of RfI in improving CLABSI outcomes, there is interest from other hospital-acquired condition teams in applying RfI to their improvement work. Thus, further studies observing the effectiveness of RfI outside of CLABSI are warranted.

## DISCLOSURE

The authors have no financial interest to declare in relation to the content of this article.
